# The Role of Stress Proteins in Haloarchaea and Their Adaptive Response to Environmental Shifts

**DOI:** 10.3390/biom10101390

**Published:** 2020-09-29

**Authors:** Laura Matarredona, Mónica Camacho, Basilio Zafrilla, María-José Bonete, Julia Esclapez

**Affiliations:** Agrochemistry and Biochemistry Department, Biochemistry and Molecular Biology Area, Faculty of Science, University of Alicante, Ap 99, 03080 Alicante, Spain; lauramata1996@gmail.com (L.M.); camacho@ua.es (M.C.); basilio.zafrilla@ua.es (B.Z.); mjbonete@ua.es (M.-J.B.)

**Keywords:** haloarchaea, stress, universal stress proteins

## Abstract

Over the years, in order to survive in their natural environment, microbial communities have acquired adaptations to nonoptimal growth conditions. These shifts are usually related to stress conditions such as low/high solar radiation, extreme temperatures, oxidative stress, pH variations, changes in salinity, or a high concentration of heavy metals. In addition, climate change is resulting in these stress conditions becoming more significant due to the frequency and intensity of extreme weather events. The most relevant damaging effect of these stressors is protein denaturation. To cope with this effect, organisms have developed different mechanisms, wherein the stress genes play an important role in deciding which of them survive. Each organism has different responses that involve the activation of many genes and molecules as well as downregulation of other genes and pathways. Focused on salinity stress, the archaeal domain encompasses the most significant extremophiles living in high-salinity environments. To have the capacity to withstand this high salinity without losing protein structure and function, the microorganisms have distinct adaptations. The haloarchaeal stress response protects cells against abiotic stressors through the synthesis of stress proteins. This includes other heat shock stress proteins (Hsp), thermoprotectants, survival proteins, universal stress proteins, and multicellular structures. Gene and family stress proteins are highly conserved among members of the halophilic archaea and their study should continue in order to develop means to improve for biotechnological purposes. In this review, all the mechanisms to cope with stress response by haloarchaea are discussed from a global perspective, specifically focusing on the role played by universal stress proteins.

## 1. Introduction

Over the years, organisms have survived a wide range of changing conditions such as temperature, salinity, oxygen levels, pH, nutrients, radiation, pressure, and toxic chemicals [1]. These agents, known as stressors, alter the status and homeostasis of the cells in all three domains of life [2]. Cell stressors can be classified by their nature: physical (heat, several types of irradiation, pressure, high salinity, sound), oxidative (H_2_O_2_, reactive oxygen species (ROS), anaerobiosis to aerobiosis shift), pH (alkalinity and acidity), osmotic (changes in the concentration of osmolytes), nutritional (starvation of carbon, nitrogen or phosphate), antibiotics (tetracycline, puromycin), alcohols (ethanol, methanol, butanol, propanol, octanol), metals (cadmium, copper, zinc, lead, nickel, tin, aluminum, chromium), insecticides (lindane, diazinon, paraquat), mechanical (compression, shearing), and others (benzene, phenol, mutagens, ammonia, arsenite, arsenate, amino acids, desiccation, anesthetics) [3]. Some organisms have been able to develop different strategies that have allowed them to adapt to these stress conditions. This has led to a great variety of habitats spread over the world existing today that are optimal for a wide range of organisms, but uninhabitable for others organisms [1]; for example, high salinity acts as a stressor for a given organism but at the same time can act as an optimal growth condition for another organism [2]. It is well known that the central event caused by a stressor on the cell is protein denaturation [4]. However, microorganisms have developed other molecular adaptation mechanisms to each stressful condition such as overexpression of stress genes or downregulation of nonstress genes. 

All three domains of life are exposed to environmental stresses that usually cause a modification in their growth. Today, the effects of abiotic stresses over cells are studied under controlled growth conditions in the laboratory using not only standard methods, but also omics techniques [4,5]. However, it is difficult to perform this type of study under natural conditions because organisms found in their natural ecosystems may grow under different simultaneous stresses, e.g., salinity and heat or drought and salinity, consequently, stress responses are usually multifactorial and integrated. Over the years, biological systems have developed mechanisms to respond in an appropriate way to natural environmental stresses that can end up damaging the DNA and proteins in cells. In order to study the response to a defined experimental perturbation, system approaches allow the formulation of a hypothesis that simulates cycles of perturbations and analysis to verify them [6].

In ecological niches and habitats, microbial life has started to be affected due to increased anthropogenic activity as well as extreme changes in the ecosystems as a result of global warming. In fact, the majority of organisms are not able to deal with rapid changes because they need optimal conditions to maintain their growth, while prokaryotes like extremophiles are able to adapt and survive under extreme conditions [7]. An example of extremophiles are halophilic organisms like Haloarchaea that belong to the Archaea domain and require at least 1.5 mol/L of sodium chloride to survive and grow [8]. These microorganisms mainly inhabit saline and hypersaline ecosystems and therefore may be exposed to changes in salinity due to rain and water runoff that would have the effect of diluting the normal salt content. It should be noted that these saline environments are strongly influenced by climate variability and extreme weather events. For the time being, there is no accurate data on their whole extension in the world, but the incidence and extent of these hypersaline areas are increasing due to the effects of climate change and anthropogenic activities such as desertification [9]. Not only are halophilic Archaea exposed to salinity stress, but also to ultraviolet (UV) radiations, high ionic stress, high temperature, and alkaline pH [7]. Needless to say, climate change is predicted to lead to more frequent and severe stressful events such as heavy rainfall or an increase in temperature [10,11,12]. Therefore, it is essential to analyze the effects of climate change conditions on microbial communities and understand how these communities recover cell homeostasis after their exposure to different stress conditions [13]. 

Focusing on the Haloarchaea, this review provides an overview of the proteins and biomolecules involved in their environmental stress responses. Knowledge about how and when haloarchaeal stress genes are expressed and how they respond to these adverse stimuli is less advanced than in other group of prokaryotes. Transcription stress genes and their regulation mechanisms are still unclear and, in general, very little is known about these genes in comparison with those from bacteria and eukaryotes. Thus, the present work represents an excellent basis to further advance not only the study of the Archaea domain under stress conditions, but also in the field of biotechnology, as it could find applications of industrial (protein production or design of stress resistant microorganisms) or environmental interest (global warming). 

## 2. Stress in Halophilic Archaea 

The central characteristic of hypersaline environments (soda lakes, lagoons, saltern ponds, salt deposits, artificial solar salterns, submarine salt domes, surface salt deposits and salt marshes) is the high concentration of total salt found in water and soil higher than 3.5% (*w*/*v*) of seawater [14,15,16,17]. These saline and hypersaline environments are found throughout the world; where both saline/hypersaline environments and semi-arid areas are concomitant, drylands cover about 41% of the Earth’s total land surface [18]. Extreme halophiles (salt-loving organisms) found in all three branches of life (Bacteria, Archaea, and Eukarya) inhabit these natural environments. Within the Archaea domain, the most salt-requiring microorganisms are extreme halophiles belonging to the Haloarchaea class [19]. Members of this class stand out for being obligate halophiles, but also for being facultative aerobes, heterotrophic, slightly thermophilic, and phototrophic [17]. Moreover, haloarchaea require a salt concentration of 10–35% (*w*/*v*) for optimal growth and constitute the dominant microbial population when the salt concentration exceeds 16% (*w*/*v*) [15,16]. In order to withstand this high salinity without losing protein structure and function, these microorganisms have developed different molecular adaptations, such as a high density of acidic residues on the surface of haloarchaeal proteins and a decrease in exposed hydrophobic residues through loss of surface lysine [20,21,22]. These negative charges on the surface form clusters that bind networks of hydrated ions, which prevent protein precipitation in the presence of a high cytoplasmic KCl concentration. In contrast, an absence of salt promotes unfolding of halophilic proteins due to repulsive forces between negative charges of acidic amino acids [14,15,16,20] 

From an ecological point of view, there are well known effects due to the high content of salts, whereby the overall biodiversity decreases as halotolerant and halophilic species develop and replace halosensitive biota [23]. The properties of the salterns are stable from year to year, so the gradient of salinity remains relatively constant, which allows communities of halophilic and halotolerant organisms to adapt to local salinity [24]. Nonetheless, many haloarchaea species thrive despite a wide variety of environmental stresses (Table 1), to which they exhibit considerable tolerance, making them interesting from an ecological perspective. As they have developed strategies enabling them to grow and proliferate under a multitude of extreme conditions, some haloarchaea species are considered “polyextremophiles” [17]. At the time of this review, *Halobacterium* sp. NRC-1 is being used as a model organism for the study of tolerance mechanisms to environmental stress agents due to its extremophilic character as well as it also being widely distributed in nature.

Since members of *Haloarchaea* thrive in extremely saline environments, one of the potential problems to address is osmotic stress due to desiccation and dilution. For instance, rainfall and evaporation may cause major changes in salinity, and halophilic Archaea have to adapt their metabolism in order to survive. Consequently, they have mastered the art of osmoadaptation to salt at the gene and protein levels [49]. These natural hypersaline environments are characterized by low water activity due to high NaCl concentrations, so microorganisms living there must develop mechanisms to prevent water loss by osmosis to avoid protein inactivation, inhibition, and even denaturation. Basically, there are two broad strategies, “salt in” and “low salt in” used to resist osmotic stress and achieve osmotic stability by halophilic microorganisms in hypersaline environments [50]. The “salt in” effect is commonly used by Haloarchaea and consists of compensating ion levels: KCl usually provides an osmotic balance. This accumulation of dominant ion K^+^ and also of Na^+^ within cells is maintained by ion pumps and protein transport [50,51]. By contrast, the most used strategy in the Bacteria domain, “low salt in” is used to produce or accumulate compatible osmotic solutes at low concentration in the cytoplasmic medium, such as sugar derivates, polyols, betaines, thetines, amino acids, glutamine amide derivates, and ectoines that have osmotic potential. Unusually, the genera *Natronobacterium* and *Natronococcus* accumulate as solutes 2-sulphotrehalose and glycine, respectively [50,52,53]. This is one of the few examples of Haloarchaea that use the “low salt in” strategy, with the accumulation of intracellular KCl being the most common strategy in the Archaea domain. *Halobacterium* sp. NRC-1 is one of the most well-known systems. It has several genes that code for multiple active K^+^ transporters and an active Na^+^ efflux system involved in the maintenance of the intracellular ionic concentrations appropriate for growth [54,55]. 

Temperature in high-salinity environments can vary widely, both daily and seasonally, and is considered another relevant limiting factor in the survival of many halophilic microorganisms [44,56]. In order to maintain physiological balance under challenging temperature conditions, Haloarchaea must withstand these changes. In the case of the haloarchaeon *Halobacterium* sp. NRC-1, its exposure to extreme temperatures elicits specific responses to promote the protein folding and limiting factors responsible for growth inhibition [25]. Indeed, this haloarchaeon showed induction of molecular chaperones, which are a diverse group of proteins that assists in the folding/unfolding and assembly/disassembly of other macromolecular structures [25,57,58]. On the other hand, in response to cold temperatures, *Halobacterium* sp. NRC-1 changes the expression of cold shock proteins, alters polar lipids biosynthesis, and increases the expression gas vesicles [25]. These mechanisms that adjust differential gene expression are used to maintain homeostasis as environmental conditions change.

Haloarchaea have been isolated from saline environments with a broad pH range, from extremely acidic lakes such as Lake Afrera, Ethiopia [59] to environments with a very high pH, such as Lake Magadi, Africa and Mono Lake, California [60,61]. However, little is known about the molecular adaptations associated with the growth of haloarchaea at extreme pH values. One of the few studies performed on haloarchaea described the transcriptomic profile differences of *Halorubrum lacusprofundi, Haloferax volcanii,* and *Halobacterium* sp. NRC-1 linked to extreme pH values. This work concludes that alkaline conditions promote, in the three species analyzed, the overexpression of genes involved in stress, such as the *hsp20* family, *uspA,* or *groEL* chaperone. This response is quite similar to that described in other prokaryotes belonging to the Bacteria domain. However, under acidic conditions, *Hrr. lacusprofundi* presents a different transcriptomic profile to *Hfx. volcanii* and *Halobacterium* sp. NRC1. In particular, transcripts related to spore formation and dormancy are up-regulated in *Hrr. lacusprofundi*, which suggests that this microorganism has a species-specific response and becomes dormant at an acidic pH [34].

Some halophilic habitats contain a high concentration of metals, since various anthropogenic activities like urbanization and industrialization contribute to heavy metal pollution of these sites [62]. Such heavy metal pollution is an important environmental issue due to the toxic effects of metals, and their accumulation leads to health and ecological problems [63]. Therefore, haloarchaea have developed different metal-resistance mechanisms (extracellular metal sequestration by biopolymers, metal efflux mediated by specific transporters and enzymatic detoxification) to withstand stress from transition metals such as arsenic, cadmium, copper, cobalt, zinc, and iron [39,64], which allow them to tolerate a broad range of these metal concentrations [42,43,64]. For instance, in *Halobacterium sp. NRC-1,* has developed two different arsenic resistant mechanisms. In the first one, arsenic is transported out of the cell by promoting the expression of a cluster of genes *(arsADRC*) encoding enzymes that alter the metal oxidation state and, in the other, arsenic is converted to methylated species like dimethylarsinate (DMA), trimethylarsine oxide (TMAO), or trimethylarsine (TMA) gas thanks to *arsR2M* operon [65,66].

Although temperature, pH, and salinity, as previously mentioned, are potential stressors, oxygen is also considered a relevant stressor. Halophilic archaea generally grow under aerobic conditions in hypersaline environments, despite possessing anaerobic capabilities. The poor concentration of oxygen in hypersaline environments strictly regulates anaerobic metabolism in order to allow organisms to thrive in their natural environment. Under low oxygen conditions, some members of halophilic archaea such as *Halobacterium salinarum* and *Haloferax mediterranei* produce gas vesicles to enable cells to float vertically and reach the oxygen-rich surface layers in the water column, where the oxygen concentration is higher to prevent oxygen limitation and sustain aerobic growth [45,67,68]. However, this strategy is highly limited, depending on external factors such as light, salt concentration, temperature, and oxygen availability. However, under strict anaerobic conditions, some halophilic archaea species like *Hfx. mediterranei* are able to use alternative electron acceptors, such as nitrate, dimethyl sulfoxide (DMSO), trimethylamine *N*-oxide (TMAO), chlorate, and perchlorate, as a strategy to maintain a respiratory metabolism that supports cell growth [46,47,48,69]. On the other hand, *Halobacterium* sp. NRC-1 was found to grow on either DMSO or TMAO, and, to some extent, fumarate, as the terminal electron acceptor, but with nitrate no significant growth was observed [70,71]; no growth was detected in others like *Haloquadratum walsbyi* due to anaerobic respiration [72]. 

Recent studies have explored the tolerance of extremely halophilic archaea to high levels of sunlight in the natural environment. As halophilic archaea are found in saltern crystallizer ponds, they are exposed to intense solar radiations, which damage their DNA. In fact, haloarchaea are considered the second most resistant microorganism worldwide, only slightly less resistant than *Denicoccus radiodurans* [73]. Under these conditions, halophilic archaea, apart from the mechanism for repairing DNA damage (photoreactivation, nucleotide excision repair, base excision repair, and homologous recombination), have developed different strategies for the tolerance, survival, and maintenance of their growth that provide protection from the consequences of habitual exposure to intense UV. These strategies include the synthesis of carotenoids, oxidative damage avoidance, polyploidy, and a genome with high CG content [74]. Photoreactivation is likely to be the most important strategy for dealing with UV irradiation [75]. *Halobacterium* sp. NRC-1 is highly resistant to the effects of UV because it undergoes efficient photoreactivation of DNA damage; for that reason, it has been used as a model organism in a wide variety of studies on tolerance to UV and ionizing radiation tolerance [74,75,76,77]. It is remarkable that *Halobacterium* sp. NRC-1 is resistant to high UV radiation withstanding up to 110 J/m^2^ without losing its viability [6]. Not only can *Halobacterium* sp. NRC-1 tolerate UV radiation; it can also resist gamma radiation due to its ability to repair double-strand DNA breaks [73,78]. 

## 3. Response to Stress in Halophilic Archaea

Microorganisms achieve their physiological functions and survive only if they have proteins that are synthesized in optimal concentrations and folded in a correct three-dimensional configuration, that is to say, a native configuration [4]. By contrast, there are some occasions when proteins lose their functional configuration and become denatured. This process may in some cases be reversible, as mentioned above, with partial protein unfolding and loss of function being the major effects of a stressor [79,80]. Thus, the stress response counteracts the effects caused by denatured proteins or the process of avoiding protein denaturation.

The stress response manifests itself in several ways: down-regulation of many housekeeping genes; activation of stress genes; partial protein unfolding; housekeeping gene repression and down-regulation; variations in cell mobility; changes in the permeability of the cell membrane to some compound; cell aggregation which results in multicellular structures of different complexity; and other phenomena in terms of changes in electrolytes or other components [4,80]. Some of the above mentioned events may occur in response to stressful conditions; however, the down-regulation of many housekeeping genes and the activation of stress genes are the most important strategies for cells under stress conditions [79]. 

There are several strategies to protect the cell against stressors in the domain Archaea: stress proteins, chaperones, thermoprotectants, the proteasome, multicellular structures, and other molecules whose function is not fully understood [80]. All these components are useful in order to ensure tolerance, macromolecular repair, and cell homeostasis maintenance. Indeed, the knowledge of the molecular mechanisms involved in the regulation stress genes of halophilic archaea that thrive under stressful conditions is fundamental. These studies are relevant for the exploration of ecosystems that are not habitable to humans, mammals, or plants. In addition, some recent research suggests that haloarchaeal species like *Hfx. volcanii* and *Halobacterium* sp. NRC-1 may be a candidate species for life on Mars as well. This possibility is being considered since haloarchaea are resistant to extreme conditions, and evidence suggests that chloride and sulfate salts are abundant on Mars [81]. 

### 3.1. Heat Shock and Stress Proteins

In general terms, an important component of the stress response is the activation of stress genes, increasing their transcriptional activity in order to protect the cell. However, not only are stress genes actively transcribed under non-physiological normal conditions, but also they have transcriptional activity in the absence of stress [80]. The products of stress genes against temperature are called Heat-shock response (Hsp), which can be classified according to mass. Hsp refers to a protein encoded by a gene that is induced by heat shock. Nowadays, considering the information available on stress proteins, the overwhelming majority comes from bacteria and eukaryotes, not from Archaea. Thus, it is crucial to identify the genes involved in cell survival under stress conditions in halophilic organisms, in order to improve the knowledge and use of these genes for biotechnological purposes. Furthermore, understanding stress proteins will help us to design several more efficient gene engineering strategies.

To sum up, the main known functions of stress proteins are to assist other proteins in the cell with their folding, translocation, and assembly, but also to contribute to the elimination of proteins that may endanger cell viability and have lost their usefulness [82,83,84,85]. They are also involved in some other functions, such as binding to denatured proteins [86], preventing enzymes from denaturation and aggregation [87], participation in signal transduction pathways [88], rRNA processing [89], etc. Therefore, stress proteins are considered multifunctional, ubiquitous, and promiscuous [80]. The motifs identification of these proteins helps to highlight their diversity of function.

Hsps are classified into six major conserved families according to their molecular weight (MW): Hsp100, Hsp90, Hsp70, Hsp60, Hsp40, and sHsp (small heat shock proteins). The two most studied families are Hsp70 and Hsp60, which contain 70 kDa and 60 kDa, respectively. These have mostly been studied in many bacterial and eukaryotic species. The Hsp60 family is also called chaperonins and is included within the molecular chaperones. Hsp70 (DnaK) carries out its chaperone functions by teaming up with Hsp40 (DnaJ) and GrpE in the cytosol [90,91]. This archaeal machinery is much closer to bacterial equivalents due to previous events of lateral transfer from bacteria [92], whereas, chaperonins are closely related to eukaryotic counterparts [57,58]. In addition, the chaperone machinery (DnaK, DnaJ, GrpE*)* is usually present in bacteria and eukaryotes, while some species of archaea lack it or do not have all the components of this machinery [5,92,93]. 

To have an idea of the representation of all Hsp families included in haloarchaea, in Table 2, it is shown that not all the families of Hsp are present in the halophilic archaea. The heavy heat shock protein family, Hsp100, is present neither in halophilic archaea nor in any archaeal species. The most common families in haloarchaea are Hsp70 and Hsp60. These families have been studied for years in Bacteria and Eukarya.

#### 3.1.1. Molecular Chaperones

When the cell has to respond to difficult environmental situations, stress proteins play the most important role; among them are the molecular chaperones [5]. In the domain Archaea, systems like the chaperone machine and chaperonins are better known than stress genes and proteins; in fact, the expression and regulation of the chaperonin machine have also been studied. There are extensive studies about the impact on the chaperonin gene expression under salt stress conditions. These studies conclude that chaperonins are likely to play important roles in salt tolerance, for example, in the case of the halophilic archaeon *Methanohalophilus portucalensis,* the expression profile changes in response to hyper-salt stress and hypo-salt stress [94].

Some stress proteins are chaperones (Table 2) whose main function is to help fold newly synthesized cytosolic proteins into their functional conformation, preventing misfolded protein structures during and after translation, refolding denatured proteins due to a stress in the cytosol, assisting with the modulation of the oligomeric state of protein complexes, facilitating protein degradation and translocations across biological membranes, and guiding proteins in the migration to the cells where they will reside and function [95,96,97,98,99]. It is important to keep in mind that not all stress proteins are chaperones, and not all molecular chaperones are stress proteins, as some chaperone genes are not stress inducible. Chaperones and chaperonins play an important role in the maintenance of cellular proteins at the physiological level; when there is an alteration in the native conformation of a protein, the stress response will be induced and Hsp will be activated including the chaperones and chaperonins. The halophilic archaea families that contain molecular chaperone machinery genes are summarized in Table 3. These data may change as the number of sequenced archaeal genomes increases. It should be taken into account that the components of the molecular chaperone machine are highly conserved in bacteria and also in eukaryotes, but not in archaea since many archaeal species do not have them [92,100]. Despite this, all haloarchaea families have the chaperone machinery. According to the sequenced genome of the species included in haloarchaeal families, the largest number of chaperone proteins are found in *Halorubraceae*, *Natrialbaceae,* and *Haloferacaceae*, which contain more than 60 proteins.

#### 3.1.2. Peptidyl-Propyl Cis–Trans Isomerases

Another relevant family within the Hsp is sHsp, which has a molecular mass lower than 34 kDa (Table 2). Peptidyl-propyl *cis–trans* isomerase (PPIase) is one of the members of this family. PPIase is an enzyme found in various organisms belonging to the three phylogenetic domains of life. Its function is to mediate the peptidyl-prolyl isomerization; it is a step in the folding of proteins, so it catalyzes the isomerization between the *cis* and *trans* forms of the peptide bonds [79,101]. In other words, protein folding requires rotation of the peptidyl-propyl bonds and this enzyme catalyzes this rotation making it faster [2]. PPIases are divided into three unrelated structural families: cyclophilins (Cyp), FK506 binding proteins (FKBPs), and the parvulin family [102,103]. This enzyme has been found in the genomes of all six families of haloarchaea, but there have still been no characterization studies of PPIases in any species. In archaea, there has been research into the hyperthermophilic methanarchaeon *Methanococcus jannaschii* in response to heat and cold shock, where a PPIase belonging to the FKBP family is characterized in some detail [104]. Few PPIases are found in archaeal microorganisms. Future studies will deepen our understanding of PPIases in halophilic archaea to find out how the folding machinery works in response to stress agents to maintain balanced concentrations of proteins for survival and growth.

### 3.2. Thermoprotectants

Thermoprotectants are nonprotein molecules that have the ability to protect cells against stress agents. In some organisms, trehalose plays an important role in stress tolerance and protein folding. Not only has it been shown that this disaccharide protects DNA from radiation damage, but it also protects proteins and cellular membranes from the denaturation caused by stressors [105,106]; it also helps ensure genomic integrity, as the cell fragmentation is lower. In the halophilic archaeon *Halococcus hameliensis*, trehalose led to a great improvement in solar radiation resistance. Maybe this archaeon accumulates this solute as a strategy to survive under osmotic pressure [107]; however, it is clear that in this microorganism trehalose is not directly related to DNA protection [107]. 

### 3.3. Multicellular Structures

Another way to deal with a stressor is by generating multicellular structures. However, this line of defense is only used by some archaea to fight against stressors (mechanical, physical, and chemical). Biofilms are multicellular structures that are formed in extreme and moderate environments [108]. Biofilms constitute several benefits against stress such as a source of energy and nutrients or desiccation. Biofilm-forming archaea have been observed in single archaea species or in mixed communities, with bacteria in a broad range of habitats from hydrothermal vents to Antarctic sea water [109,110,111]. As there is a wide variety of archaeal species identified in mixed biofilms, this suggests a significant ecological impact on the biogeochemical cycles of carbon, iron, sulfur, and nitrogen [112,113,114]. Biofilms are now recognized as dynamic communities in which phenotypic diversification allows microorganisms to adapt to different environments. 

This multicellular structure is found in a wide variety of haloarchaeal species from *Halobacterium, Haloferax,* and *Halorubrum*, showing two different types of biofilm structures. However, the biofilm architecture differs between the genera. The first is a carpet-like, multi-layered, biofilm containing macro- and microcolonies, present in *Halobacterium salinarum* DSM 3754^T^*,* strain R1, and a novel Antarctic isolate*, t-ADL DL24*. The second type is characterized by large aggregates of cells that adhere to surfaces, present in *Haloferax volcanii* and *Halorubrum lacusprofundi*. The presence of extracellular polymers was also revealed to stabilize them [115]. Since there are various biofilm architectures in halophilic archaea, it is questionable if these versatile architectures under artificial conditions could be present in their natural environment when there are not only single species but also mixed populations [108].

### 3.4. Other Stress Proteins

In addition to Hsps proteins, there are other genes and proteins that should be considered pertinent to the stress response because of: the increasing amount of protein and/or mRNA during or after stress, the high concentration of the protein during stress, assistance with protein folding, and a similar sequence to a well-known stress gene or protein [80]. These other stress proteins found in archaea species are exemplified by DNA repair proteins, proteasome, or survival proteins as universal stress proteins (USP) (Table 4). These proteins have been identified by bioinformatic analysis in sequenced haloarchaeal genomes in the six families of haloarchaea that is to say, they are present in *Halococcaceae, Halorubraceae*, *Natrialbaceae, Haloferacaceae*, *Haloarculaceae,* and *Halobacteriaceae.* A list of proteins identified under stress conditions in the haloarchaea group is given in Table 4. 

#### 3.4.1. DNA Repair Proteins

In halophilic archaea, DNA repair mechanisms of UV-induced damage include photoreactivation, nucleotide excision repair, base excision repair, and homologous recombination [121]. Within the group of proteins that helps in DNA repair, DNA mismatch proteins have been found to have the function of correcting the mispaired or unpaired bases from replication errors, physical damage to bases, and homologous recombination, increasing the overall fidelity of DNA synthesis [122]. By studying the response of the halophilic archaeon *Methanohalophilus portucalensi* after long exposure to salt stress, the expression of MutS, a mismatched DNA repair protein, was detected [94]. Otherwise, the UvrABC system has the function of catalyzing the recognition and processing of DNA lesions. In haloarchaea, it was found that the genes *uvrA, uvrB,* and *uvrC* are required to repair UV photoproducts in *Halobacterium* sp. NRC-1 and that this pathway is exclusively responsible for the excision repair of UV lesions from its genome [116]. To sum up, these are some examples, but there are a great number of DNA repair proteins used by halophilic archaea to protect against external environmental stresses. These stresses involving DNA repair not only occur in UV radiation but also in osmotic stress, low water activity, and desiccation.

#### 3.4.2. Proteasome

There is still some doubt about the biological roles of the archaeal proteasome, but it should be considered under the umbrella of stress. The proteasome is considered to be the main molecular machine for regulating the degradation of intracellular proteins. Selective protein degradation plays an important role in the cellular physiology [123]. Therefore, the elimination of molecules that are abnormal because they tend to aggregate, or precipitate is carried out by proteases. This degradation of proteins includes those that have been mistranslated, misfolded, damaged or are malfunctioning. Meanwhile, there are two key pathways in eukaryotes for the selective removal of undesirable proteins. These pathways are the ubiquitin-proteasome system and autophagy-lysosome system [124,125]. In contrast, the existence of any pathway has not been confirmed in either bacteria or archaea. Proteasomes and small ubiquitin-like modifier proteins have been reported, but their functions and induction mechanisms have not yet been elucidated [123]. As a result, the complexity of the eukaryotic proteasome is contrasted with the relative simplicity of its archaeal counterpart; in archaea there are only two types of α and β-subunits, while in eukaryotes there are at least 14 unique subunits [123]. A proteasome is a multi-subunit protease complex synthesized in cells whose function is involved in the degradation of proteins that are partially or fully unfolded due to stress or an alteration like a mutation [79]; its catalytic activity is to cleave the peptide bonds with high specificity. A proteasome is required for cell growth due to its role in protein degradation. 

There is little information about the proteasome concerning halophilic archaea; but the proteasome system in *Hfx. volcanii* has been widely studied in the presence of some stressors (high temperature, low salt, and the addition of l-canavanine), making it the best studied organism in haloarchaea [126,127]. This research concludes that *psmA* and *panA* are required to withstand low salt stress and l-canavanine, while *psmA* helps to overcome the hypersensitive responses of high temperature stress. *psmA* and *panA* genes encode α1 and PanA proteasomal proteins, which are predominant during the growth of this haloarchaeon [119,120]. 

#### 3.4.3. Survival Proteins

Another important family of proteins, which are significantly overexpressed under unfavorable environmental stresses, is the universal stress protein (USP). These proteins are induced under heat/cold shock, nutrient starvation, oxidative stress, heavy metal toxicity, exposure to cycloserine, ethanol, antibiotics, etc. [128]. This family of stress proteins is clarified in the following section.

#### 3.4.4. Transcriptional Factors

The growth and survival of archaeal organisms depend on their ability to deal with environmental variations, unexpected stress conditions, and metabolic changes. An important mechanism underlying this response to stress is the gene regulation mediated by transcriptional factors (TFs). Archaeal TFs are classified into families based on their structural similarity. The most abundant families in archaea are Lrp/AsnC, MarR, ArsR, and TrmB, according to their members per genome [129]. As TFs play an important role in the adaptation to environmental and nutritional changes to preserve the survival of organisms, this section will show some examples of TFs studied in haloarchaea involved in the stress response. It is clear that not all the TFs play a role in stress conditions.

In general, the Lrp/AsnC family may be involved in several functions: the regulation of amino acid metabolism and transport, the maintenance of processes (translation and DNA repair), and the regulation of central and energy metabolism. In the case of the haloarchaeon *Hbt. salinarum*, it has been shown that transcriptional factors Lrp/AsnC are regulated in response to oxidative stress conditions [130]. Otherwise, the ArsR/SmtB TFs family has different functions, including leading under stressful conditions. In bacteria, these ArsR-like TFs, which have been previously characterized, have been shown to be linked to stress-inducing concentrations of heavy metals by repressing the expression of operons [131]. Unlike TFs in bacteria, archaeal ArsR-like TFs have also developed several physiological roles and signal responses [132]; an example of this is found in the *Methanothermobacter thermautotrophicus* as it has been demonstrated that an ArsR-like MsvR regulates the expression of genes involved in oxidative stress response; this suggests that it may play a role in detecting the redox state of the cell [133]. 

## 4. Universal Stress Proteins among Three Domains of Life

The Universal Stress Proteins (USP) superfamily was discovered in *Escherichia coli*; since then, a large number of USPs have been identified in bacteria, plants, fungi, protozoa, and archaea, but not in humans, making USPs potential drug targets [134,135]. The induction of these proteins is due to the growth arrest caused by a wide range of biotic and abiotic stresses. Therefore, these proteins are expressed in a large variety of conditions, such as heat shock, genotoxicity, membrane damage, and starvation of carbon, nitrogen, sulphate, phosphate, and amino acids [128,136,137,138]. Under stressful conditions, USPs are overexpressed by different mechanisms that help the organism to survive [139]. USPs are also known to help pathogens with the invasion of host organisms [140]. USPs cause survival defects under a variety of growth-arrested conditions in *Mycobacterium tuberculosis* [135], *Salmonella typhimurium* [141], and *Burkholderia pseudomallei* [142], making an important contribution to virulence and highlighting the importance of stress resistance regulation in pathogenicity, and survival within the host.

This group of proteins is directly or indirectly involved in transport functions, protein scaffolding, and signaling [143]. Furthermore, USPs appear to be linked to resistance to DNA-damaging agents and thus protect their nucleic acids from external stresses [128].

Multiple paralogs of USPs have been found; the number of copies depends on the organism. *E. coli* harbors six different proteins, USPA, USPC, USPD, USPE, USPF, and USPG, which have shown undefined functions ranging from oxidative stress resistance to motility [144]. These six *E. coli* USPs are classified into four subclasses based on the amino acid sequence homology and structural similarity: Class I includes USPA, USPC, and USPD without ATP binding motif; Class II comprises USPF and USPG with ATP binding motif; and USPE has two tandem-repeated USP domains in a polypeptide, designated as E1 and E2, which are grouped into Class III and Class IV, respectively [145]. Each subclass has its own specific function to deal with a particular environmental stress. Both USPA and USPD play an important role in the defense against oxidative stress, and USPD also participates in iron metabolism. As well as their anti-oxidative function, USPC and USPE are involved in cellular motility, cell adhesion, and cell aggregation. In addition to the functions of USPC and USPE, USPF and USPG play different roles in cell migration, negatively regulating mobility, and positively controlling cell agglomeration. These results clearly suggest that bacterial USPs have developed distinct physiological functions in order to act in a coordinated way, defending the cell from an external stress. Although their functions have been demonstrated by their structural diversity, the biochemical and molecular mechanism remains largely unknown [144,145]. Focusing on the molecular structures of the diverse bacterial USPs, the USP domain is always present, followed by another catalytic motif in some cases. These motifs include amino acid permease, protein kinase, Na^+^/H^+^ antiporter, and a voltage gated Cl^−^ channel [128,139]. 

Similar to bacterial USPs, all the USPs identified in plant species contain at least one USP domain and one other catalytic motif. Hence, plant USPs play a distinctive role in specific tissues and developmental stages under diverse stress conditions [145]. The other catalytic motifs found in plant USPs are the cation exchanger, the C1 motif of Insensitive to Killer toxin3 (IKI3), the HomeoDomain leucine zipper (HDzip), SWI2/snf2 and Mudr (SWIM)-zinc finger, tyrosine kinase, and serine/threonine kinase [145]. As in bacterial USPs, the physiological and biochemical functions are still unknown but are essential to plant development and growth.

**Figure 1:**
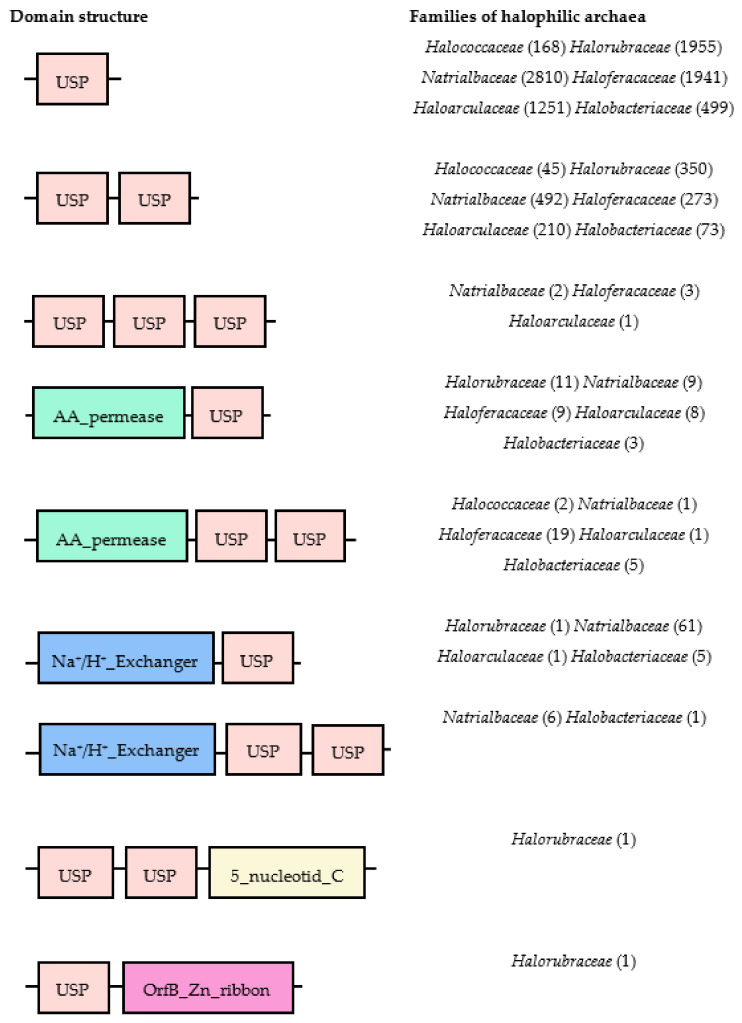
Domain structures of halophilic archaeal Universal Stress Proteins (USPs). The diverse domains were obtained from the UniProt database (https://www.uniprot.org/). The numbers in parenthesis indicate the numbers of USPs having this specific structure in each haloarchaea family. Each type of domain is in different color-boxes. AA: amino acid.

Like the bacterial and plant USPs, archaeal USPs have different functional motifs. The USPs in the six families of halophilic archaea *Halococcaceae, Halorubraceae*, *Natrialbaceae, Haloferacaceae, Haloarculaceae,* and *Halobacteriaceae* have been analyzed using different bioinformatic tools (Figure 1). The molecular structures of the diverse halophilic archaeal USPs only contain a USP domain or USP domain fused with other catalytic motifs, such as amino acid permease or a Na^+^/H^+^ exchanger. A single USP motif is the most common domain structure in all families and contains around 140–160 amino acids. These motifs are not conserved in all halophilic archaea, so the combination of the USP domain with other highly divergent functional motifs might produce multiple functions to protect the organism against harsh conditions.

The genome analysis of the USPs found in all available genera of halophilic archaea is summarized in Table 5, but the rest that do not appear in this table, *Halomicroarcula, Halosiccatus, Halocalculus, Halomicrococcus, Halorubellus, Halorussus, Halostella, Salinirubrum, Salinirussus, Halegenticoccus, Halopelagius, Haloparvum, Halovarius, Natribaculum, Natronobiforma,* and *Saliphagus*, had no USPs in their genome. Given the large number of halophilic archaeal USPs, it is reasonable to think that proteins play crucial roles in diverse aspects of metabolism to maintain cell homeostasis. Some genera comprise a large number of USPs, such as *Halorubrum, Haloferax,* and *Haloarcula*, which present a total of 1939, 1301, and 970, respectively. The analysis of protein sequences revealed that all these USPs proteins contain at least one USP domain. As previously explained, in the case of plants and bacterial USPs, halophilic archaeal USPs have several catalytic motifs that will determine numerous functions derived from their variety of structural characteristics. It is strongly suggested that halophilic archaeal USPs have a great functional diversity. During the evolutionary process, the pressure against stress may lead to the fusion of the USP domain with other catalytic motifs, providing the archaea with the ability to protect them from external environmental stress. This is exemplified by the fusion of the USP domain with the amino acid permease, which allows the transport of amino acids into the cell. The Na^+^/H^+^ exchange motif, fused with the USP domain, sends sodium out of the cell, preventing its accumulation. Therefore, under stressful conditions, these proteins are involved in multiple reactions and diverse cellular processes. Not only is it necessary to determine their functional specificity, but also to discover their physiological and biochemical functions for archaeal metabolism. Unravelling these functions may unlock new biotechnological applications of these proteins. 

Furthermore, throughout history, these climatic changes have occurred many times and they are an evolutionary engine to adapt to stress in order to colonize new habitats.

## 5. Conclusions

This review highlights critical factors that influence the survival of microorganisms, in general, and of halophilic archaea, in particular, under different stress conditions that remain largely unknown. Interactions among haloarchaea in saline/hypersaline environments can be influenced by temperature, pH, UV radiation, concentration of heavy metals, etc. These stressful conditions are increasing daily due to climate change, and microorganisms have had to adapt to them throughout history to colonize new habitats. Thus, new biotechnological and industrial applications may be unlocked after discovering the functions of stress proteins, focusing on USPs. USPs are emerging as important players in stress resistance. Hence, the results of this review should serve as the basis for future investigations to elucidate the role of stress proteins in resistance against stressors from a molecular and biochemical point of view. Within this context, new questions arise: How and when do stress genes respond to a given stress? How are these responses regulated? How many molecules are involved in this response? Can we define gene regulatory networks? How will microorganisms adapt to the effects of climate change in their ecosystems? How do antistress mechanisms work at the molecular and genetic levels? Many questions remain unanswered, but this review is a good starting point. Undoubtedly, more work could result in interesting biotechnological applications in order to improve the capacity of microorganisms to withstand environmental shifts.

## Figures and Tables

**Table 1:** Examples of the most studied stressors tested in halophilic archaeal cells.

Stressor	Species	Reference(s)
Hypo-osmolarity	*Halobacterium* sp. NRC-1*, * *Haloferax mediterranei, * *Haloferax volcanii, Halolamina sp*	[25,26,27,28,29,30]
Hyper-osmolarity	*Halobacterium* sp. NRC-1*,* ** *Haloferax mediterranei,* *Haloferax volcanii, Halolamina* sp.	[25,26,27,28,29,30,31]
Ultraviolet radiation	*Halococcus hamelinensis,* *Halobacterium* sp. NRC-1*, Haloferax volcanii*	[6,32,33]
pH	*Haloferax volcanii, Halorubrum lacusprofundi, * *Halobacterium* sp. NRC-1	[34]
H_2_O_2_	*Halobacterium* sp. NRC-1*, Haloferax volcanii*	[35,36,37,38]
Heavy metals	*Halobacterium* sp. NRC-1*, Halomicrobium mukohataei, Halococcus salifodinae, * *Haloferax volcanii, Haloferax mediterranei, * *Haloarcula japónica, Halorubrum* sp., *Halobacterium cutirubrum*	[39,40,41,42,43]
Temperature	*Halobacterium* sp. NRC-1 *Haloferax volcanii*	[25,30,44]
Oxygen	*Halobacterium* sp. NRC-1*, * *Haloferax mediterranei,* *Haloferax denitrificans, Haloferax gibbonsii,* *Haloarcula marismortui, Haloarcula vallismortis*	[45,46,47,48]

**Table 2:** Classification of Heat-shock response (Hsp) according to molecular mass.*.

Family Name	Subunit (kDa)	Families of Haloarchaea
Hsp100 Heavy Hsp, high MW	≥100	-
Hsp90	81–99	*Halorubraceae, Natrialbaceae, Haloferacaceae, Haloarculaceae,* *Halobacteriaceae*
Hsp70 Chaperones	65–80	*Halococcaceae, Halorubraceae,* *Natrialbaceae, Haloferacaceae,* *Haloarculaceae, Halobacteriaceae*
Hsp60 Chaperonins	55–64	*Halococcaceae, Halorubraceae,* *Natrialbaceae, Haloferacaceae,* *Haloarculaceae, Halobacteriaceae*
Hsp40 DnaJ	35–54	*Halococcaceae, Halorubraceae,* *Natrialbaceae, Haloferacaceae,* *Haloarculaceae, Halobacteriaceae*
sHsp Small Hsp	≤34	*Halococcaceae, Halorubraceae,* *Natrialbaceae, Haloferacaceae,* *Haloarculaceae, Halobacteriaceae*
Others (Proteases, etc.)	various	*Halococcaceae, Halorubraceae,* *Natrialbaceae, Haloferacaceae,* *Haloarculaceae, Halobacteriaceae*

* Adapted from [80]. Protein numbers were obtained from the UniProt database (https://www.uniprot.org/).

**Table 3:** Molecular machinery (Hsp70, Hsp 40 and GrpE proteins) found in some halophilic archaea *.

	Number of Proteins		
Haloarchaea Families	Hsp70(DnaK)	Hsp40(DnaJ)	GrpE
*Halococcaceae*	6	5	6
*Halorubraceae*	66	63	69
*Natrialbaceae*	67	62	64
*Haloferacaceae*	62	60	62
*Haloarculaceae*	46	44	48
*Halobacteriaceae*	24	22	23

* Protein numbers were obtained from the UniProt database (https://www.uniprot.org/). They were only considered proteins whose genes were identified as *dnaK, dnaJ,* and *grpE,* and not those where the protein names are prefoldin alpha and prefoldin beta classified as *pfdA* and *pfdB,* respectively.

**Table 4:** Examples of stress genes involved in protecting the cell from unfavorable external conditions, identified in halophilic archaea.

Gene(s)	Protein	Theoretical Function	Species	Ref.
*mutS*	DNA mismatch	This protein is involved in the repair of mismatches in DNA.	*Methanohalophilus portucalensis*	[94]
*uvrA* *uvrB* *uvrC*	UvrABC system	The UvrABC system catalyzes the recognition and processing of DNA lesions.	*Halobacterium* sp. NRC-1	[116]
*radA*	DNA repair	Involved in DNA repair and in homologous recombination.	*Haloferax volcanii*	[117]
*ligA* *ligN*	DNA ligase	Essential for DNA replication and repair of damaged DNA.	*Haloferax volcanii*	[118]
*psmA* *psmB* *psmC* *panA* *panB*	Proteasome subunit	Involved in protein degradation.	*Haloferax volcanii*	[119,120]

**Table 5:** Number of Universal Stress Proteins (USPs) found in halophilic archaea *.

Genus	Number of USPs	Genus	Number of USPs
*Halapricum*	26	*Haloplanus*	244
*Haloarcula*	970	*Haloprofundus*	51
*Halomicrobium*	118	*Haloquadratum*	106
*Halorhabdus*	40	*Halobaculum*	21
*Halorientalis*	139	*Halobium*	22
*Halosimplex*	32	*Halohasta*	38
*Natronomonas*	197	*Halolamina*	88
*Haladaptatus*	148	*Halonotius*	105
*Halalkalicoccus*	83	*Halopenitus*	59
*Halanaeroarchaeum*	25	*Halorubrum*	1939
*Halarchaeum*	47	*Salinigranum*	52
*Haloarchaeobius*	31	*Halobiforma*	184
*Halobacterium*	412	*Halopiger*	165
*Halodesulfurarchaeum*	21	*Halostagnicola*	107
*Halomarina*	21	*Haloterrigena*	428
*Halovenus*	28	*Halovivax*	60
*Natronoarchaeum*	30	*Natrarchaeobius*	174
*Salarchaeum*	17	*Natrialba*	352
*Halococcus*	278	*Natrinema*	571
*Halobellus*	165	*Natronobacterium*	289
*Haloferax*	1301	*Natronococcus*	205
*Halogeometricum*	194	*Natronolimnobius*	362
*Halogranum*	160	*Natronorubrum*	557
*Halopelagius*	70	*Salinarchaeum*	32

* The number of USPs was obtained from UniProt database (https://www.uniprot.org/).
